# Thermal Stability and Non-Isothermal Kinetic Analysis of Ethylene–Propylene–Diene Rubber Composite

**DOI:** 10.3390/polym15081890

**Published:** 2023-04-14

**Authors:** Huda Alfannakh, Nisrin Alnaim, Sobhy S. Ibrahim

**Affiliations:** 1 Department of Physics, College of Science, King Faisal University, P.O. Box 400, Al-Ahsa 31982, Saudi Arabia; 2 Department of Physics, Faculty of Science, Cairo University, Giza 12613, Egypt

**Keywords:** EPDM composite, borax, TGA analysis, Kissinger–Akahira–Sunose, Friedman, Flynn–Wall–Ozawa, Augis–Bennett/Boswell, iso-conversional

## Abstract

The purpose of this study was to investigate the thermal stability and the decomposition kinetics of ethylene–propylene–diene monomer (EPDM) composite samples loaded with and without lead powder (50, 100, and 200 phr lead) using thermogravimetric analysis (TGA). TGA was carried out at different heating rates (5, 10, 20, and 30 °C/min) under inert conditions in the temperature range of 50–650 °C. Lead addition did not significantly change the onset temperature or peak position corresponding to the maximum decomposition rate of the first derivative of the TGA curve (DTGA) (onset at about 455 °C and T_m_ at about 475 °C). Peak separation for the DTGA curves indicated that the main decomposition region for EPDM, the host rubber, overlapped the main decomposition region for volatile components. The decomposition activation energy (E_a_) and pre-exponent factor (A) were estimated using the Friedman (FM), Kissinger–Akahira–Sunose (KAS), and Flynn–Wall–Ozawa (FWO) iso-conversional methods. Average activation energy values of around 231, 230, and 223 kJ/mol were obtained for the EPDM host composite using the FM, FWO, and KAS methods, respectively. For a sample loaded with 100 phr lead, the average activation energy values obtained via the same three methods were 150, 159, and 155 kJ/mole, respectively. The results obtained from the three methods were compared with results obtained using the Kissinger and Augis–Bennett/Boswell methods, and strong convergence was found among the results of the five methods. A significant change in the entropy of the sample was detected with the addition of lead powder. For the KAS method, the change in entropy, ΔS, was −3.7 for EPDM host rubber and −90 for a sample loaded with 100 phr lead, α = 0.5.

## 1. Introduction

EPDM is one of the most popular rubbers due to its many advantages and properties. This type of rubber is utilized in the preparation of composite materials with additives and ingredients. It is also used for the preparation of blended rubber composites with another type of rubber or polymer such as NBR, PVC, etc., to acquire new characteristics and open wide horizons for technological applications. This type of rubber is famous for its radiation shielding properties, especially against gamma rays and neutrons [1,2,3,4]. This is due to its hydrogen abundance. EPDM composites are also used in thermal insulation, leakage prevention, and other relevant applications. Additionally, composites based on EPDM can be made for electromagnetic shielding using additives with magnetic and electrical properties [5,6].

The thermal stability and decomposition mechanisms for polymeric and rubber composites are among the key properties of materials exposed to high temperatures. In view of the exposure of many products based on EPDM rubber to a heating environment, such as reactors, the automobile industry, etc., it is important to study the effect of any additives on the thermal stability of the composite, as well as to predict the most common degradation mechanism [7,8,9,10,11].

Many studies have examined the effectiveness of blending EPDM rubber with other types of rubber to improve its physical or chemical properties; for instance, blending EPDM with natural rubber has been shown to improve its mechanical properties [12]. Scientists have studied the impact of additives such as accelerators [13], vulcanization agents [14], and other additives (during the preparation process) on the properties of EPDM [15,16,17]. Much research has been conducted on the thermal stability and decomposition mechanisms of EPDM composites or blends. The researchers examined how additives and other treatments affect the composite decomposition mechanisms and thermal stability.

Neelesh Ashok et al. [18] investigated the mechanical, viscoelastic, thermal, and transport properties of EPDM-CIIR blends reinforced with bis(3-triethoxysilylpropyl)tetrasulfide (TESPT)-grafted nano-silica after exposure to different cumulative radiation doses. The nanocomposites had a degradation onset temperature of 503 °C, compared to 485 °C for unfilled blends. The thermal degradation activation energy was evaluated using the Coats–Redfern and Horowitz–Metzger models. In addition, this research group performed research related to the use of EPDM in radiation protection, and they investigated the thermal decomposition of different EPDM composites [19,20,21]. A Güngör et al. [22] investigated the effect of surface-modified waste walnut shells (as a filler) on the mechanical, thermal, and rheological properties of EPDM/waste walnut shell filler composite. The TGA results showed similar T50 temperatures for the CB30 and CB30 + S30 samples. This could be attributed to the walnut shells not significantly affecting thermal stability. The thermal degradation of a blend of NR/EPDM (70:30 wt%) under anaerobic and aerobic conditions was investigated using mercapto- and thioacetate-modified EPDM (EPDMSH) [23]. The kinetic parameters of thermal degradation were evaluated from non-isothermal TGA experiments undertaken at different heating rates. The results indicated that the presence of functionalized polymers (in low proportions) led to an increase in the calculated decomposition activation energy, which indicates an increase in resistance to thermal degradation. Using thermal kinetic analysis, the kinetic parameters and decomposition mechanisms of ethylene propylene copolymers (EPM) and ethylene propylene diene terpolymers (EPDM) were also studied by Antonio Perejón et al. [24].

The thermal degradation of all compositions has been described using the random scission model, and it has been shown that the activation energy for the thermal decomposition of EPM decreases with an increase in the proportion of propylene. The EPDM chemical composition is not strongly correlated with the activation energy for pyrolysis.

The thermal decomposition mechanisms of the host EPDM rubber composite and of the same composite loaded with lead powder have not been studied in a detailed manner, despite the importance of this mixture in many applications [25,26]. Therefore, the focus of this research was to study the effect of adding lead powder at different concentrations to the basic composite and to identify the decomposition mechanisms through the study of thermal stability and kinetic thermal analysis. This research also aimed to compare the different models with each other and with the iso-conversion methods, in order to maximize the knowledge outcomes from this work.

## 2. Materials and Methods

### 2.1. Preparation of EPDM Composites

The EPDM host composite and the composites loaded with varying phr of lead were synthesized by using a two-roll mill. Carbon black (N-220) and other ingredients (ZnO, stearic acid, paraffin oil, etc.) were mixed on an open rubber roll mill (ASTM D3182–16) at different concentrations of lead (50, 100, and 200 phr). After mixing, the composites were vulcanized via compression molding and hot pressed at about 153 °C, under 4 MPa, for 5 min. The final product was in the form of sheets of suitable thickness (4 mm) and diameter (10 cm) for examination.

The sample ingredients and components are outlined in Table 1. The values are expressed in phr (parts per hundred).

### 2.2. Thermogravimetric Analysis

Thermogravimetric analysis (TGA) was performed using a TA Instruments Q50 thermogravimetric analyzer, under a nitrogen (N_2_) flow of 20 mL/min. The measurements were carried out at different heating rates (5, 10, 20, and 30 °C/min) with constant mass of about 10 mg.

## 3. Theoretical Approach

### 3.1. Models for Thermal Kinetic Analysis

The decomposition or conversion rate (alpha) is used in analyzing thermal decomposition mechanisms and can be expressed as follows [27]:

(1) $$\alpha =\frac{w_{0}-w_{t}}{w_{0}-w_{f}}$$

where *w*_0_, *w_t_*, and *w_f_* are the initial, residual (at time t), and final weights of the material, respectively. The rate of conversion (*dα*/*dt*) is directly proportional to the conversion function *f*(*α*):

(2) $$\frac{d\alpha }{dt}=k\;f\left(\alpha \right)={\left(1-\alpha \right)}^{n}$$

where $\frac{d\alpha }{dt}$ is the rate of conversion in s^−1^, k is the rate constant, *α* is the degree of conversion, and *n* is the order of reaction.

In the Arrhenius equation, the activation energy is quantitatively related to the reaction rate. The equation thus provides a quantitative basis for understanding how the reaction rates depend on the activation energy. The rate constant as a function of temperature is given by

(3) $$k=A\;\exp\;(\frac{-E}{RT})$$

where *E* is the activation energy (J.mo1^−1^), *A* is the pre-exponential factor, *R* is the gas constant (8.31 J.mo1^−1^.*K*^−1^), and *T* is the absolute temperature (*K*). Using these equations, one can obtain

(4) $$\frac{d\alpha }{{\left(1-\alpha \right)}^{n}}=\frac{A}{\beta }\;\exp\;(-\frac{E}{RT})$$

(5) $$\mathrm{Let}\;g\left(\alpha \right)=\int_{0}^{\alpha }\frac{d\alpha }{{\left(1-\alpha \right)}^{n}}=\frac{A}{\beta }\;\int_{T_{0}}^{T}\exp\left(-\frac{E}{RT}\right)dT$$

The above integral cannot be solved exactly, but it can be expressed in numerical form or by some approximations. For estimating the kinetic parameters, model-free iso-conversional methods such as Friedman’s (FM), Kissinger–Akahira–Sunose (KAS) [28], and Flynn–Wall–Ozawa (FWO) [29,30] were chosen.

Using the KAS method and according to (6), the relationship between ln[β/*T*^2^] and 1/*T* was plotted for each value of conversion α. From the slope and the intersection, E_a_ and A can be calculated.

(6) $$\ln\left[\frac{\beta }{T^{2}}\right]=\ln\left[\frac{AR}{g\left(\alpha \right)}\right]-\frac{E}{RT}$$

Based on Doyle’s approximation (Doyle 1962), the integral in (5) yields the following Equation (7) via simplification, which forms the basis of further iso-conversional methods developed by Flynn and Wall (Chan and Balke 1997) and Ozawa [29,30].

(7) $$\ln\left[\beta \right]=\ln\left[\frac{AE}{R\;g\left(\alpha \right)}\right]-5.331-1.052\;\frac{E}{RT}$$

Thus, the activation energy and pre-exponential factor can be determined from the slope and intercept of the linear relation ln *β* vs. 1/*T* for constant values of *α*.

According to Friedman (1964) [31], the Friedman method (a differential iso-conversional method) provides correct values of the kinetic parameters:

(8) $$\ln\left[\frac{da}{dt}\right]=\ln\left[\beta \frac{d\alpha }{dT}\right]=\ln\left[\;A.f\left(a\right)\right]-\frac{E}{RT}$$

where ln(*β da*/*dT*) is plotted against 1/*T* at constant values of α, and from the slope and intercept, both E_a_ and A can be determined.

### 3.2. Thermodynamic Analysis

By applying the following equations with the obtained activation energy, the thermodynamic parameters, including the pre-exponential factor (*A*), changes in enthalpy (Δ*H*), Gibbs free energy (Δ*G*), and entropy (Δ*S*), can be determined [32,33]:

(9) $$A=\beta \;E\exp\left(\frac{E}{R\;T_{m}}\right)/\left(R\;T_{m}^{2}\right)$$

(10) $$\Delta H=E-R\;T_{\alpha }$$

(11) $$\Delta G=E+R\;T_{m}\;\ln\left(\frac{k_{B}T_{m}}{A\;h}\right)$$

(12) $$\Delta S=\frac{\Delta H-\Delta G}{T_{m}}$$

where *k_B_* is the Boltzmann constant, *h* is the Plank constant, *T_m_* is the temperature of the maximum decomposition rate, and *T_α_* is the temperature corresponding to *α*.

## 4. Results and Discussion

### 4.1. Thermogravimetric Analysis

**Figure 1:**
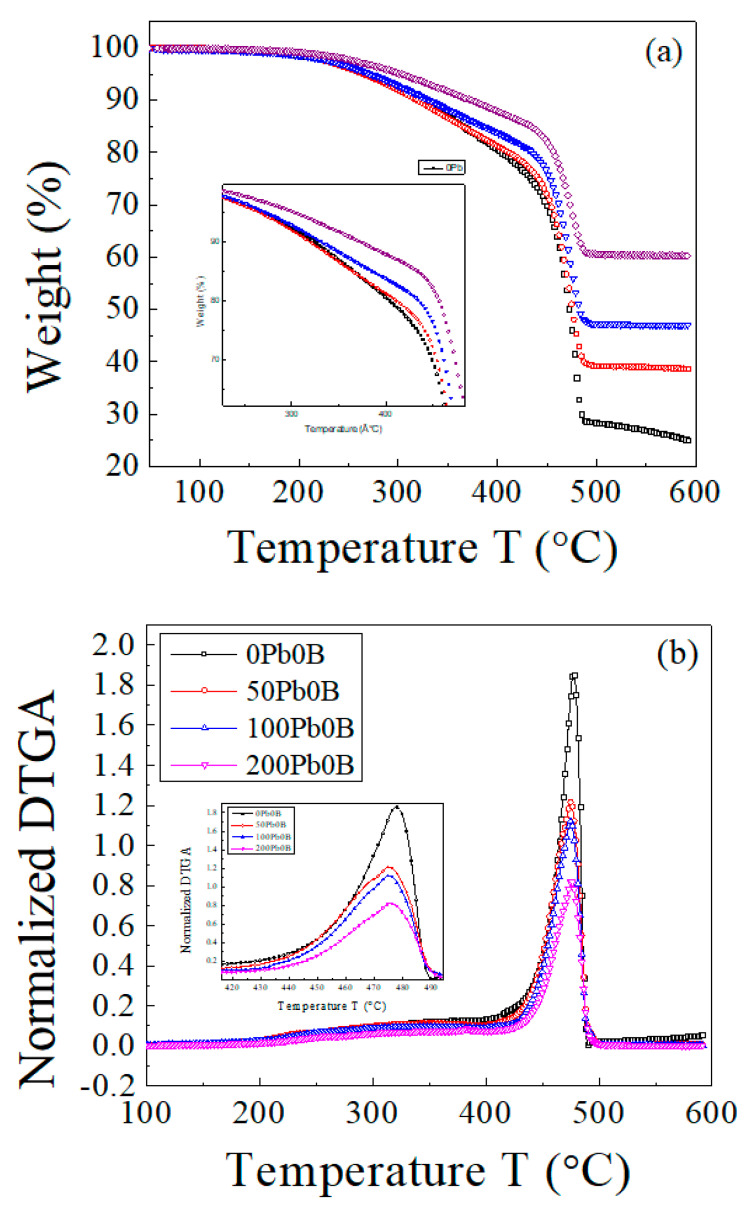
The TGA and DTGA curves of EPDM host composite (0 phr lead) and samples loaded with 50, 100, 200 phr lead. Scanning rate, 10 °C min^−1^. (**a**) TGA curves; (**b**) DTGA curves.

The TGA and DTGA curves of the EPDM host sample and samples loaded with 50, 100, and 200 phr lead are presented in Figure 1. The composite samples underwent two main decomposition stages.

**Figure 2:**
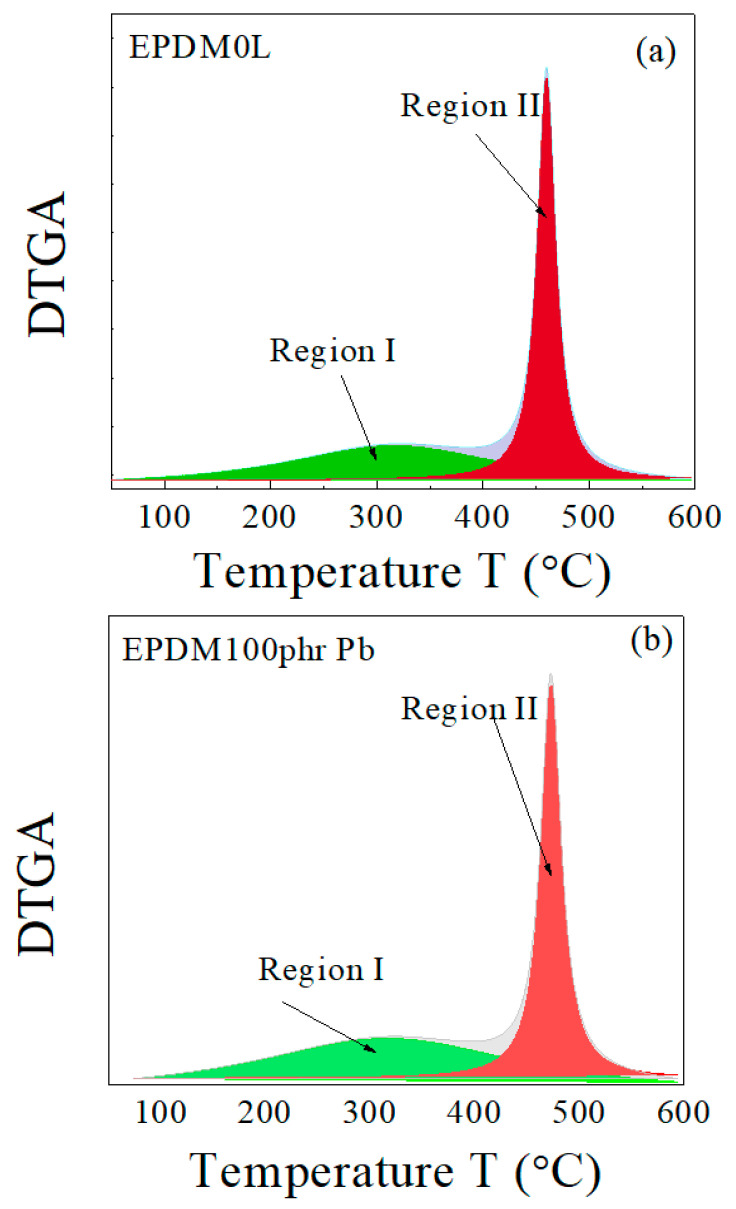
Peak separation of the DTGA curves for EPDM host samples (**a**) without lead and (**b**) with 100 phr lead.

As illustrated in Figure 2, by using the Origin−Lab program (version 8.6), the main interaction stage (in DTGA) can be resolved to two main decomposition process. The first decomposition process (Region I) starts at about 50 °C and theoretically (according to the peak profile) ends at about 600 °C, while the second process (Region II) starts at about 350 °C and ends at about 580 °C. There are no references indicating that EPDM rubber decomposes at temperatures below 350 °C [34,35,36]. Decomposition in region I can thus be attributed to the volatile components used during the preparation of the host rubber composite, such as paraffin oil. Comparing the peak position of the maximum decomposition rate for region II with the literature confirms that the decomposition in this region is caused by the decomposition of the host rubber, EPDM.

The curves clearly indicate that as the concentration of lead powder in the samples increases, the sample becomes more thermally stable, and the residual mass increases [26]. As a result of the addition of lead, the position of maximum decomposition (≈277.5 to 275 °C) did not change significantly, indicating that the influence of lead on thermal behavior is physical rather than chemical.

Table 2 lists some of the parameters that could be extracted from the thermal decomposition curves for the EPDM composite samples.

It can be seen from the above analysis that the introduction of lead powder to the EPDM host composite had an important influence on the thermal stability of the host composite. The results can be summarized as follows:

- The effect of lead powder on the main decomposition peak (maximum decomposition rate) indicates that there was no chemical interaction between lead and EPDM. The variations in these peak positions are within the experimental error. Through previous research, we note that some additives may not affect position the maximum decomposition rate of EPDM rubber [37,38].
- Lead powder’s effect is evident between 200 and 400 °C, especially at higher concentrations.
- The decomposed mass at T > 250 °C decreased with increasing lead concentration. This can be attributed to the following: Lead particles absorb a large amount of thermal energy and, in turn, delay the decomposition of the host composite. During the preparation and vulcanization process, the lead increases the thermal homogeneity of the sample, leading to the formation of a homogenous network of cross-linked chains. There has been some evidence that conductive fillers impact EPDM’s thermal behavior, especially its thermal conductivity [39,40,41].
- As the lead powder concentration increased, the residual mass increased [42].

### 4.2. Kinetic Analysis

**Figure 3:**
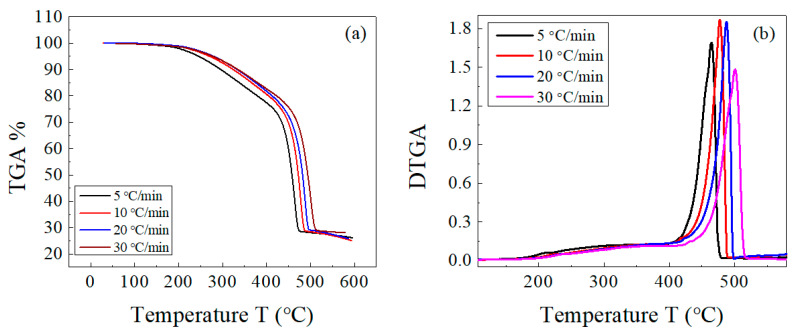
(**a**) TGA and (**b**) DTGA curves of EPDM host composite at different heating rates of 5, 10, 20, and 30 °C/min.

**Figure 4:**
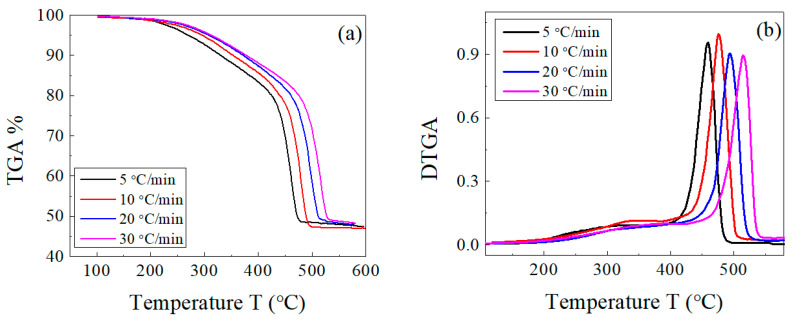
(**a**) TGA and (**b**) DTGA curves of EPDM host composite loaded with 100 phr lead powder at different heating rates of 5, 10, 20, and 30 °C/min.

The decomposition of both the host composite and the composites loaded with lead takes place in two stages: a main one for EPDM rubber [43] and a weak one for the volatile matter and other ingredients. There is an overlap between both decompositions in a range of temperatures (as previously shown in Figure 2). This behavior was not affected by change in the heating rate for both sample types, as shown in Figure 3 and Figure 4. In Figure 3b and Figure 4b, the peak corresponding to the maximum decomposition rate shifted to a higher temperature with the heating rate. Based on these data, the kinetic parameters (E_a_ and A) were estimated for both the host composite and the composites loaded with lead.

**Figure 5:**
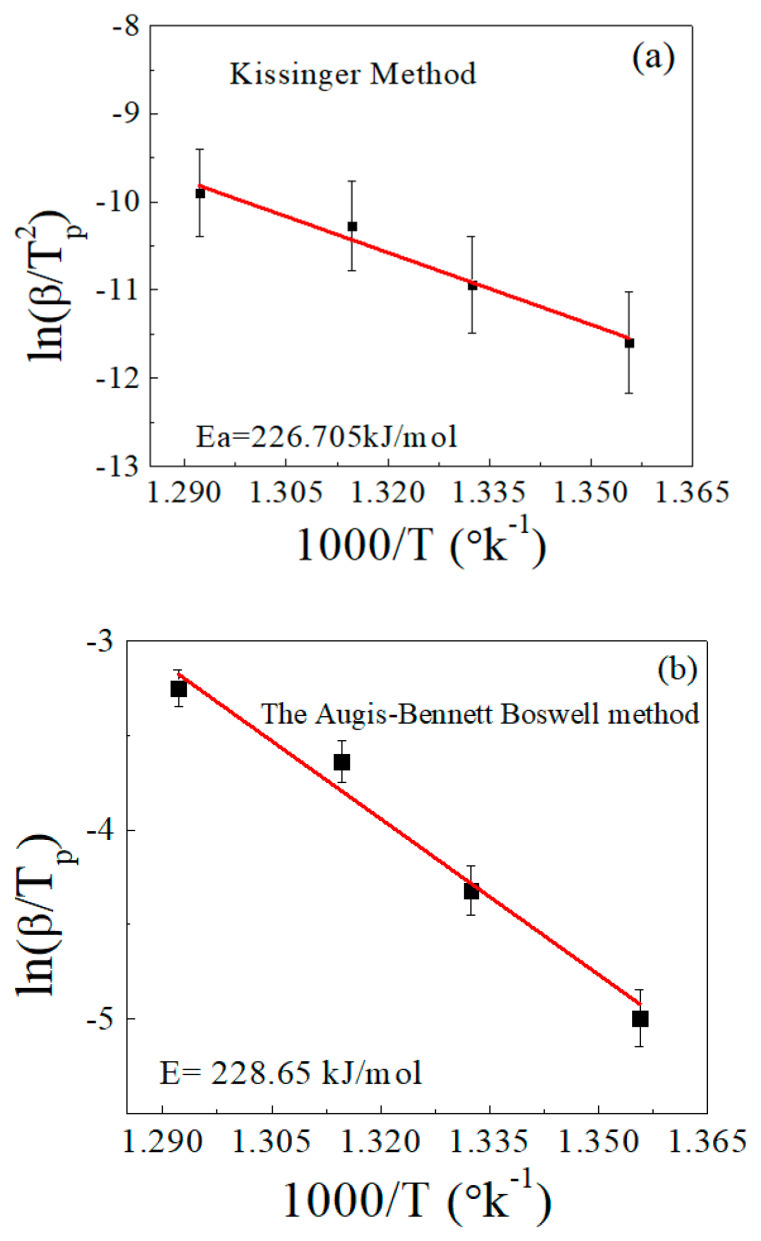
Determination of the activation energy (Ea) in accordance with (**a**) the Kissinger method and (**b**) the Augis–Bennett/Boswell method.

Figure 5 shows the relationship between ln(β/T_p_^2^) and 1000/T, as well as that between ln(β/T_p_) and 1000/T, according to the Kissinger [28] and Augis–Bennett/Boswell methods [44,45], respectively, for determination of the activation energy. Both methods gave approximately equal results within the permissible error. The activation energy value calculated using the Kissinger method was about 227 kJ/mol, while the Augis–Bennett/Boswell method gave a value of about 229 kJ/mol. The error was less than 1% between the two methods. These results are largely consistent with the references [46,47].

**Figure 6:**
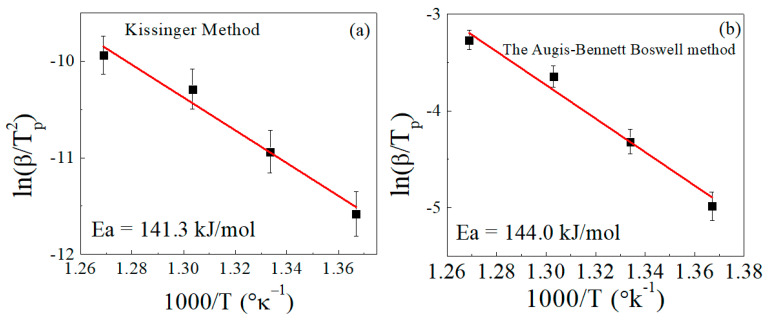
Determination of the activation energy (Ea) for EPDM/100 phr lead composite in accordance with (**a**) the Kissinger method and (**b**) the Augis–Bennett/Boswell method.

By applying the same two methods (Kissinger and Augis–Bennett), the activation energy was calculated for the sample loaded with 100 phr lead powder (Figure 6). The activation energies calculated by the Kissinger and Augis–Bennett methods were 141 and 144 kJ/mol, respectively. The addition of lead produced a significant decrease in the activation energy of decomposition, and the two methods gave values that are very close to each other. The decrease of the activation energy with the addition of lead can be attributed to the increase in the thermal conductivity of the composite sample and the decrease of entanglements in the composite matrix.

The Kissinger and Augis–Bennett methods ignore the changes in conversion rates (*dα*/*dt*) and conversion (*α*). They assume that Ea is independent of α. Three methods that are derived from (5) and account for the conversion function *f*(*α*) will be illustrated. These methods are Kissinger–Akahira–Sunose, Flynn–Wall–Ozawa, and Friedman ((6), (7), and (8), respectively).

**Figure 7:**
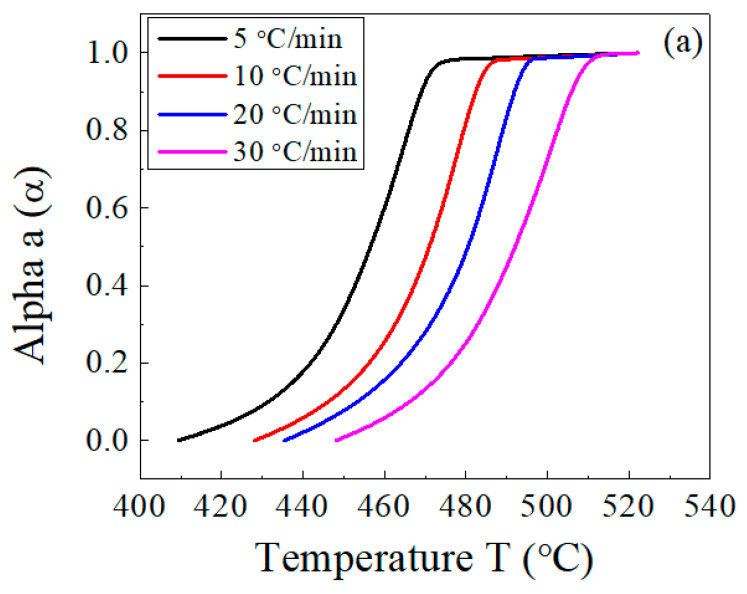

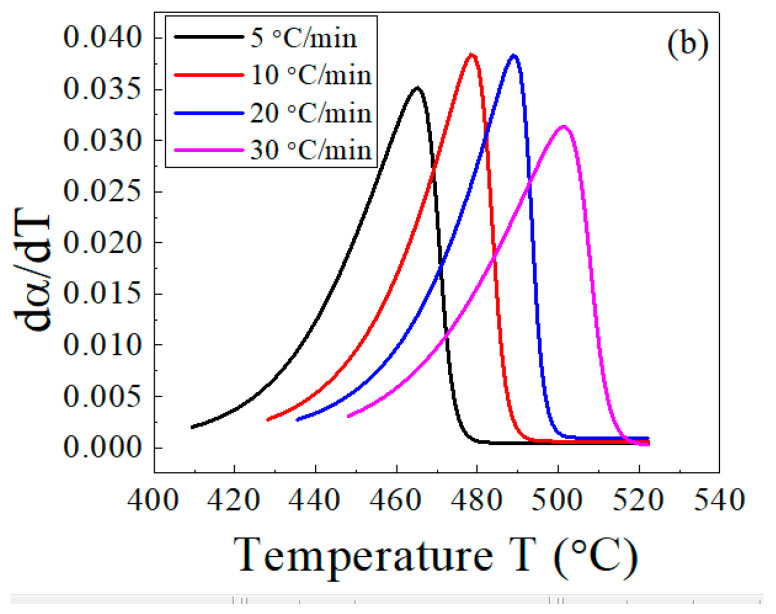
Temperature dependence of (**a**) the conversion, α, and (**b**) the conversion rate, da/dt, for the EPDM host composite sample.

Figure 7 illustrates the dependence of the conversion α (Figure 7a) and its first derivative (Figure 7b) on the temperature. Using these results, the main decomposition stage of the host EPDM with and without lead was analyzed, and a comparison among the three methods was carried out.

**Figure 8:**
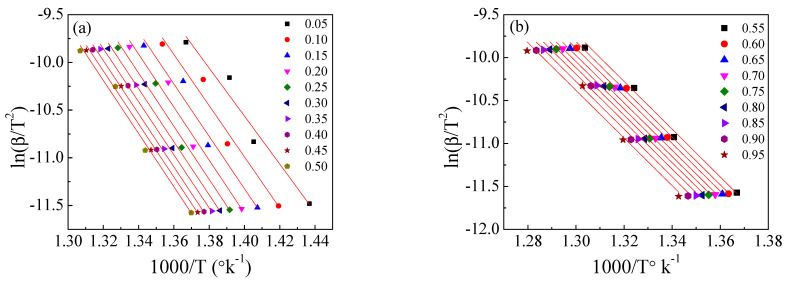

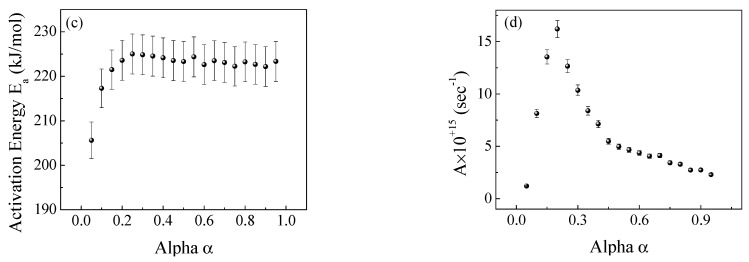
The Kissinger–Akahira–Sunose method for the EPDM host composite: (**a**,**b**) ln(β/T^2^) versus temperature; (**c**,**d**) the dependence of E_a_ and A, respectively, on conversion α.

**Figure 9:**
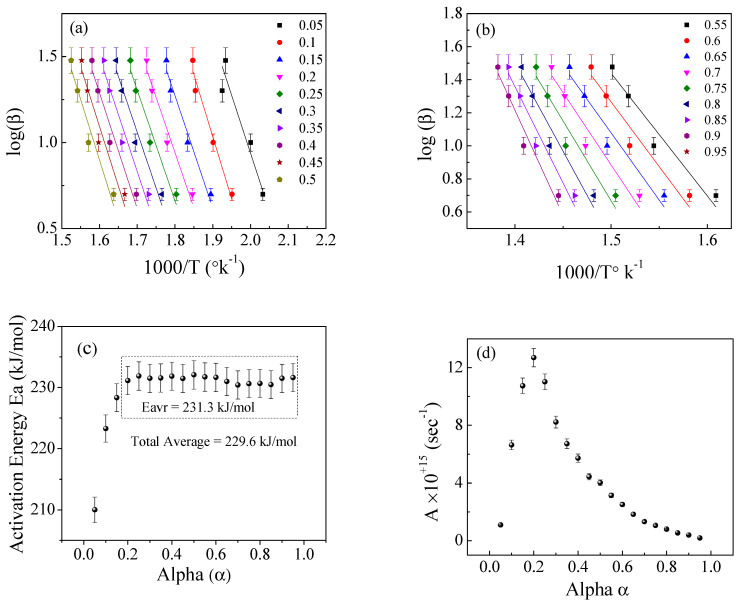
The Flynn−Wall−Ozawa (FWO) method for the EPDM host composite: (**a**,**b**) ln(β/T2) versus 1000/T; (**c**,**d**) the dependence of E_a_ and A, respectively, on conversion α.

**Figure 10:**
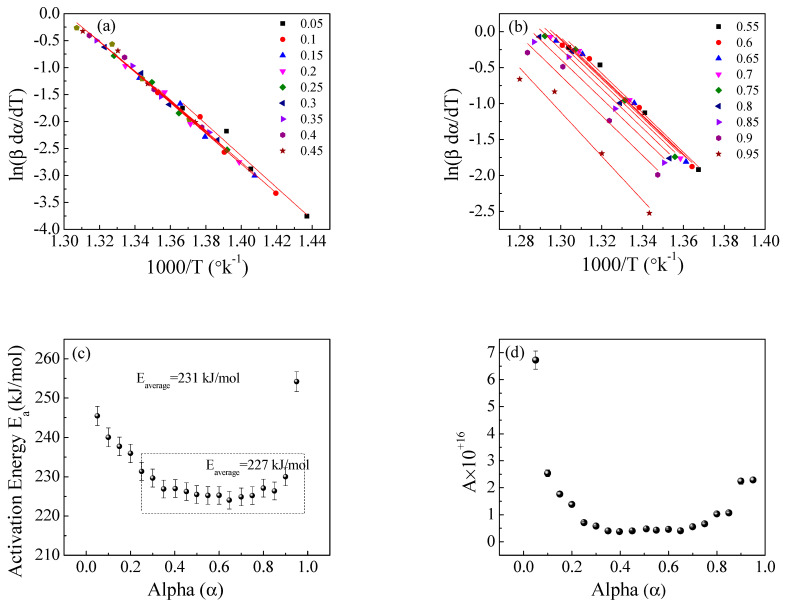
The Friedman method for EPDM host composite: (**a**,**b**) ln(βdα/dT) versus 1000/T; (**c**,**d**) the dependence of Ea and A, respectively, on α.

Figure 8, Figure 9 and Figure 10 illustrate the application of the KAS, FWO, and Friedman methods, respectively, for EPDM host composite. A resemblance was noted in the behavior of the activation energy and coefficient A for both the KAS (Figure 8c,d) and FWO (Figure 9c,d) methods. The behavior was different when applying Friedman’s method (Figure 10c,d). This may be attributed to the dependence of Friedman’s method (8) on the first derivative with respect to time of the degree of conversion (dα/dt). Despite this apparent difference in behavior, there are some common points that could be concluded. The total average values of activation energy for the three methods are distinctly close (229, 229.6, and 231 kJ/mole for the KAS, FWO, and Friedman methods, respectively). Additionally, the pre-exponential factors for the three methods are of the same order of magnitude (10^+15^) (see Figure 8, Figure 9 and Figure 10d).

In accordance with the E(α) relation for the three methods mentioned above, there is a region where the activation energy dependence on the degree of conversion (α) is weak or not significant and can be considered constant after looking at the value of its standard deviation (0.64, 0.53, and 3.31, respectively). The average activation energy for each method is close to those of the Kissinger and Augis–Bennett methods, with a slight difference not exceeding 5%, as shown in Table 3.

**Figure 11:**
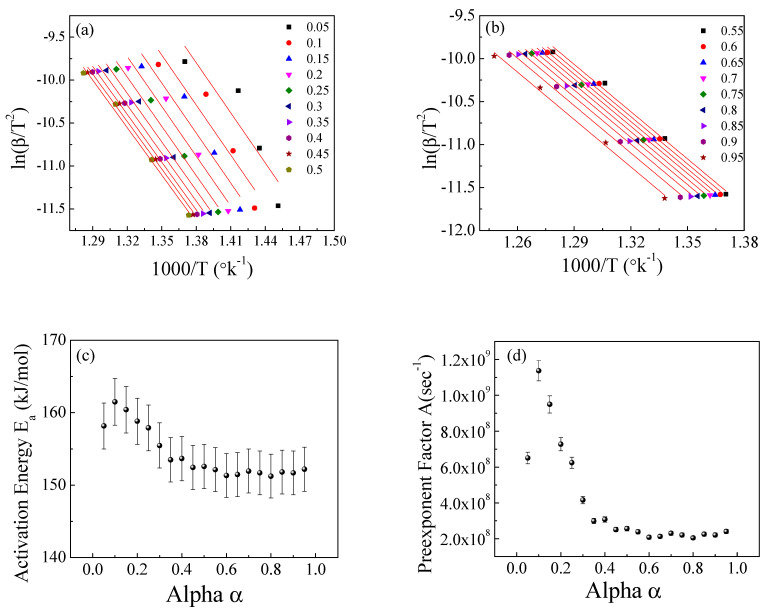
The Kissinger−Akahira−Sunose method for EPDM-100 phr lead composite: (**a**,**b**) ln(β/T2) versus temperature; (**c**,**d**) the dependence of E_a_ and A, respectively, on conversion α.

**Figure 12:**
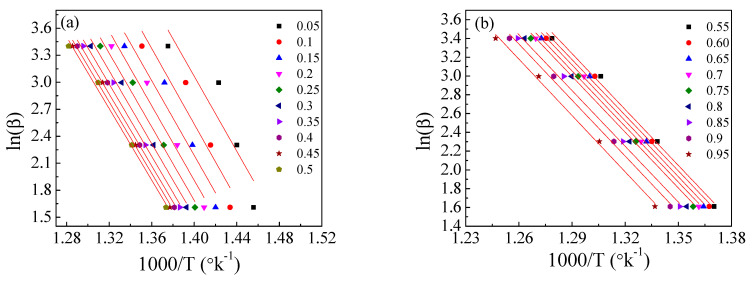

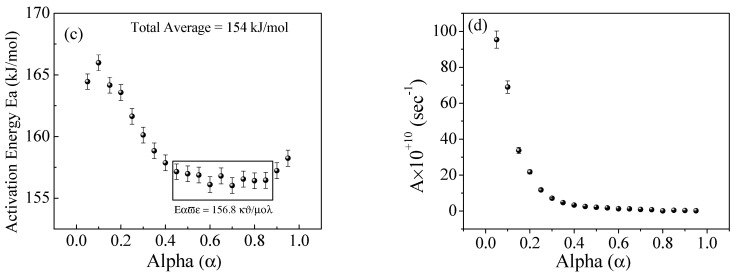
The Flynn–Wall−Ozawa (FWO) method for EPDM−100 phr lead composite: (**a**,**b**) ln(β/T2) versus 1000/T; (**c**,**d**) the dependence of Ea and A, respectively, on conversion α.

**Figure 13:**
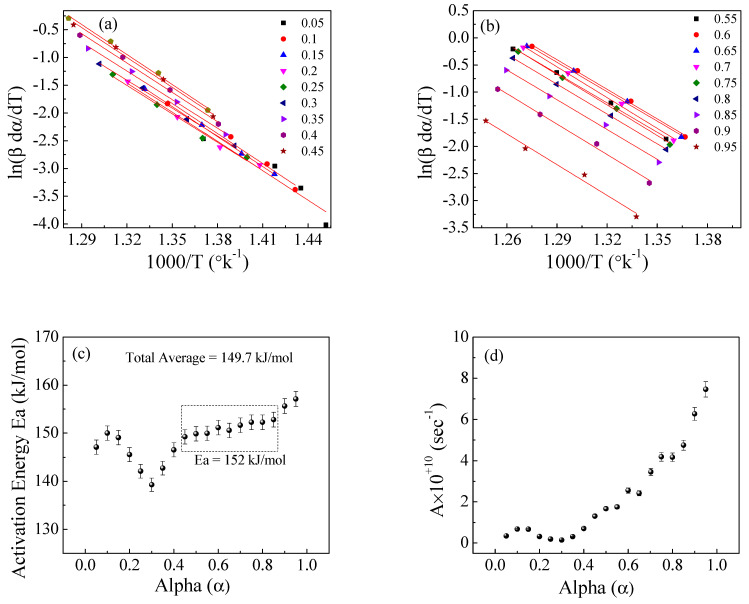
The Friedman method for EPDM−100 phr lead composite: (**a**,**b**) ln(βdα/dT) versus 1000/T; (**c**,**d**) the dependence of E_a_ and A, respectively, on conversion α.

Figure 11, Figure 12 and Figure 13 show the application of the KAS, FWO, and Friedman methods, respectively, for EPDM host loaded with 100 phr lead powder. It was observed that the activation energy values are close to each other at alpha greater than 0.4, but the activation energy changes when alpha is less than 0.4. There is no specific behavior for the three samples within this range (α < 0.4).

Table 3 summarizes the average values of activation energy over the entire range of degree of conversion, as well as the mean value of activation energy in the less dependent region (from 0.4 to 0.9) and the standard deviation for each data set.

Table 4, Table 5 and Table 6 illustrate the values of the activation energy and the pre-exponent factor, respectively (for the host composite and that loaded with 100 phr lead). The deviation in the activation energy values between the KAS and FWO methods does not exceed 4%; when compared with the Friedman method, however, the difference may reach more than 10% at some values of α. This difference may be due to the dependence of the Friedman method on the time derivative of α. The pre-exponential factor, A, varies between 10^15^ and 10^16^ for the three methods. The behavior of A is similar for the KAS and FWO methods, while it differs in the Friedman method. The fluctuation in the predicted values is larger in the case of Friedman method, as shown in Table 5.

For the three methods (KAS, FWO, and FM), (9)–(12) were used to evaluate the pre-exponential factor and thermodynamic parameters, namely, the enthalpy change (ΔH), Gibbs free energy change (ΔG), and entropy change (ΔS), based on the values of activation energy determined via the iso-conversion free-model methods [48]. Their values are listed in Table 7, Table 8 and Table 9. It is evident from these three tables that the activation energy and pre-exponential factors do not depend significantly on the conversion [49], except for small values; this indicates that the material decomposed simply and not in a complex manner. It is noted that the entropy of the rubber composite sample is much higher than the entropy of the host rubber sample (for the KAS method, ΔS was −3.7 for EPDM host rubber and −90 for a sample loaded with 100 phr lead, with α = 0.5). This result is logical, as the randomness of the system is increased by the introduction of lead particles into the host rubber system [50]. The Gibbs free energy for the EPDM composite sample is still close to the activation energy (for each alpha). This can be attributed to the fact that the nature of the rubber is still the same, and it decomposes in a simple and uncomplicated process.

The values of the pre-exponential factor (A) in Table 6 differ from those values calculated in Table 7, Table 8 and Table 9. This can be attributed to the fact that the former values depend on the value of the activation energy (calculated from the slope of the straight line) and the intercept for each value of α (according to the model or methods (6)–(8)), while the latter values (in Table 7, Table 8 and Table 9) were calculated using the general model (9) that depends on the heating rate and temperature at the maximum decomposition (T_m_) of the DTGA curve, besides other constants.

Table 10 summarizes the maximum, minimum, and average values of the basic thermodynamic parameters (enthalpy change, Gibb’s free energy change, and entropy change), activation energies (E), and pre-exponent factors (A).

**Figure 14:**
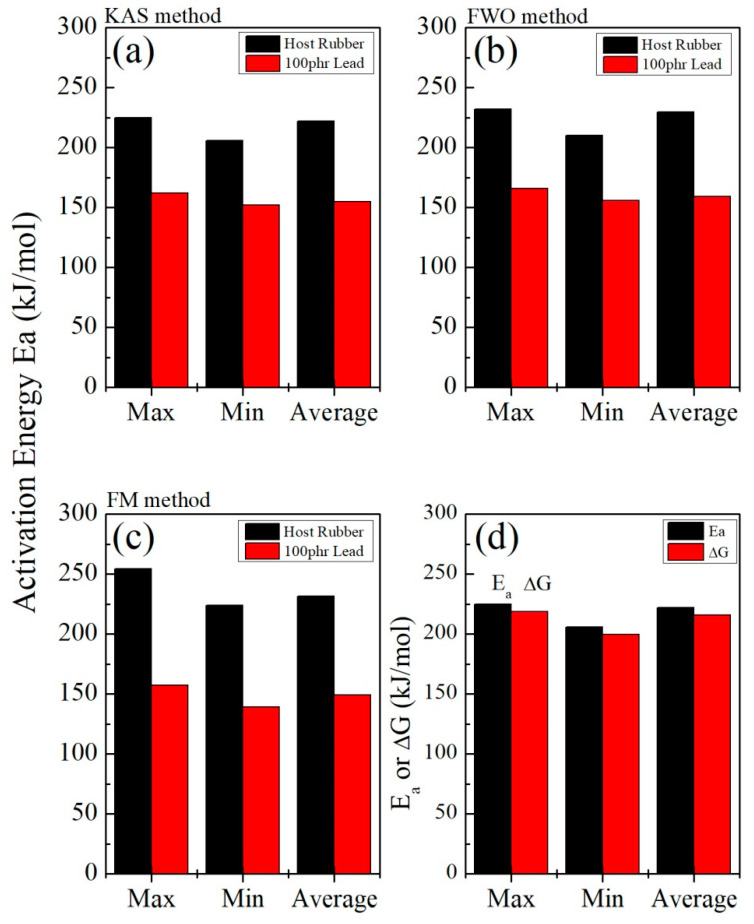
A comparison among the activation energy values of the host and lead-loaded EPDM rubber samples using the three methods (**a**) KAS, (**b**) FWO, and (**c**) FM, and (**d**) a comparison between the values of the activation energy, E_a_, and the change in the Gibbs free energy, ΔG (for KAS, using the host rubber sample as an example).

Figure 14 shows great convergence and agreement among the three methods (KAS, FWO, and FM), as well with the two other methods (the Kissinger and Augis–Bennett/Boswell methods (Figure 5)). Based on the KAS method, for example, Figure 14d shows the scope of convergence between the activation energy and Gibbs free energy change in the host sample. There is a high degree of harmony in this reaction, suggesting that it is an uncomplicated process.

## 5. Conclusions

The thermal stability of ethylene–propylene–diene monomer (EPDM) composite samples loaded with and without lead powder (50, 100, and 200 phr lead) was examined via thermogravimetric analysis (TGA) at a 10 C/min heating rate. Lead addition did not significantly change the onset temperature or peak position corresponding to the maximum decomposition rate of DTGA (onset at about 455 °C and Tm at about 475 °C). Through peak separation of the DTGA curves, it was confirmed that some degradation processes overlapped with the EPDM decomposition peak. Kinetic thermal analysis was conducted for the host sample and a sample loaded with 100 phr lead. The TGA measurements were carried out at different heating rates (5, 10, 20, and 30 °C/min). Average activation energy values of around 231, 230, and 223 kJ/mol were obtained for the EPDM host composite using the Friedman (FM), Flynn–Wall–Ozawa (FWO), and Kissinger–Akahira–Sunose (KAS) methods, respectively. Using 100 phr lead as a sample, the average activation energy values obtained via the three methods were 150, 159, and 155 kJ/mole, respectively. There was strong convergence between the results obtained via these three methods and the results obtained via the Kissinger and Augis–Bennett/Boswell methods. A significant change in the entropy of the sample was detected with the addition of lead powder.

## Figures and Tables

**Table 1:** The chemical formulations and ingredients used for sample preparation (in phr).

Material	Role	Amount (phr)
EPDM	Rubber base	100 phr
Carbon Black (N–220-(Degussa))	Reinforcement (filler)	50 phr
Paraffin Oil (ZMTH-Egypt)	Softener (plasticizer)	50 phr
Stearic acid (Behn Mayer)	Activator	2 phr
ZnO (Zink Egypt)	Accelerator	5 phr
ZDMC * (Behn Mayer)	Accelerator	3 phr
Sulfur (Polychem-egypt)	Curing agent	3 phr
Lead powder (Pb-Indiamart)	additive reinforcing filler	Variable (50, 100, 200)

* ZDMC (Zinc dimethyldithiocarbamate).

**Table 2:** Thermogravimetric analysis results of EPDM composites.

Sample	T_onset1_ °C	T_onset2_ °C	T_max2_ °C	M_65_%	m@450 °C (%)	m@550 °C (%)	M_res_
EPDM0L	239.4	455.8	477.5	458.0	69.9	27.0	24.6
EPDM50L	237.4	455.2	475.3	461	72.3	39.0	38.6
EPDM100L	235.4	453.7	475.0	467.8	76.5	47.1	47.0
EPDM200L	252.8	454.8	475.9	479.7	82.5	60.5	60.3

**Table 3:** The average activation energy values for the EPDM host composite.

	E_avr_ (α from 0.05 to 0.95)		E_avr_ (α from 0.2 to 0.8)	
	E_avr_ (kJ/mol)	STD	E_avr_ (kJ/mol)	STD
Kissinger	226.7	-	226.7	-
Augis–Bennett/Boswell	228.7	-	228.7	-
KAS	229.2	5.26	231.0	0.64
FWO	229.6	5.15	231.4	0.55
Friedman	231.0	8.20	227.3	3.31

**Table 4:** The average activation energy values for EPDM host composite loaded with 100 phr lead.

	E_avr_ (α from 0.05 to 0.95)		E_avr_ (α from 0.4 to 0.95)	
	E_avr_ (kJ/mol)	STD	E_avr_ (kJ/mol)	STD
Kissinger	141.3	-	141.3	-
Augis–Bennett/Boswell	144.0	-	144.0	-
KAS	155.0	3.6	152.7	0.5
FWO	159.0	3.3	156.8	0.7
Friedman	149.2	4.5	151.6	2.8

**Table 5:** The activation energy values calculated using three methods for the host composite (0 phr lead) and composite loaded with 100 phr lead.

α	EPDM Host			EPDM/100 phr Lead		
Alpha	KAS	FWO	Freidman	KAS	FWO	Freidman
0.05	209.07	210.00	245.46	161.54	164.46	147.10
0.10	222.91	223.29	240.06	161.46	165.98	150.00
0.15	228.12	228.34	237.75	161.27	164.16	149.08
0.20	231.00	231.15	235.96	160.56	163.57	145.56
0.25	231.71	231.88	231.38	158.89	161.64	142.11
0.30	231.29	231.52	229.67	156.50	160.13	139.29
0.35	231.30	231.57	226.89	154.53	158.85	142.74
0.40	231.56	231.86	227.00	154.18	157.87	146.55
0.45	231.14	231.49	226.23	152.99	157.15	149.24
0.50	231.72	232.08	225.46	152.91	156.97	149.86
0.55	231.33	231.73	225.24	152.51	156.87	149.97
0.60	231.21	231.65	225.28	152.51	156.09	151.14
0.65	230.49	230.99	224.04	152.84	156.80	150.56
0.70	229.87	230.43	224.87	152.36	156.02	151.65
0.75	230.06	230.63	225.21	152.85	156.55	152.28
0.80	230.08	230.67	227.12	152.49	156.41	152.28
0.85	229.86	230.48	226.39	152.31	156.45	152.81
0.90	230.89	231.50	230.01	152.90	157.23	155.64
0.95	230.98	231.62	254.20	152.88	158.24	157.10

**Table 6:** The pre-exponential factor calculated using three methods for the host composite (0 phr lead) and composite loaded with 100 phr lead.

α	EPDM Host			EPDM/100 phr Lead		
Alpha	KAS	FWO	Freidman	KAS	FWO	Freidman
0.05	1.20 × 10^15^	1.09 × 10^15^	6.73 × 10^16^	4.52 × 10^11^	9.53 × 10^11^	3.43 × 10^9^
0.10	8.14 × 10^15^	6.64 × 10^15^	2.54 × 10^16^	2.58 × 10^11^	6.89 × 10^11^	6.74 × 10^9^
0.15	1.35 × 10^16^	1.07 × 10^16^	1.77 × 10^16^	1.70 × 10^11^	3.37 × 10^11^	6.69 × 10^9^
0.20	1.62 × 10^16^	1.27 × 10^16^	1.39 × 10^16^	1.08 × 10^11^	2.17 × 10^11^	3.16 × 10^9^
0.25	1.27 × 10^16^	1.10 × 10^16^	7.05 × 10^15^	6.08 × 10^10^	1.17 × 10^11^	1.87 × 10^9^
0.30	1.04 × 10^16^	8.22 × 10^15^	5.80 × 10^15^	3.16 × 10^10^	7.06 × 10^10^	1.40 × 10^9^
0.35	8.40 × 10^15^	6.72 × 10^15^	4.00 × 10^15^	1.85 × 10^10^	4.60 × 10^10^	3.08 × 10^9^
0.40	7.14 × 10^15^	5.73 × 10^15^	3.77 × 10^15^	1.45 × 10^10^	3.24 × 10^10^	7.06 × 10^9^
0.45	5.48 × 10^15^	4.44 × 10^15^	4.03 × 10^15^	1.00 × 10^10^	2.43 × 10^10^	1.31 × 10^10^
0.50	4.97 × 10^15^	4.02 × 10^15^	4.77 × 10^15^	8.39 × 10^9^	1.99 × 10^10^	1.67 × 10^10^
0.55	4.66 × 10^15^	3.14 × 10^15^	4.27 × 10^15^	6.62 × 10^9^	1.65 × 10^10^	1.76 × 10^10^
0.60	4.37 × 10^15^	2.51 × 10^15^	4.58 × 10^15^	5.55 × 10^9^	1.22 × 10^10^	2.56 × 10^10^
0.65	4.06 × 10^15^	1.83 × 10^15^	4.05 × 10^15^	4.86 × 10^9^	1.13 × 10^10^	2.42 × 10^10^
0.70	4.12 × 10^15^	1.32 × 10^15^	5.53 × 10^15^	3.63 × 10^9^	8.05 × 10^9^	3.46 × 10^10^
0.75	3.43 × 10^15^	1.06 × 10^15^	6.65 × 10^15^	3.08 × 10^9^	6.85 × 10^9^	4.19 × 10^10^
0.80	3.29 × 10^15^	7.92 × 10^14^	1.03 × 10^16^	2.18 × 10^9^	1.18E × 10^6^	4.17 × 10^10^
0.85	2.73 × 10^15^	5.35 × 10^14^	1.07 × 10^16^	1.46 × 10^9^	3.48 × 10^9^	4.75 × 10^10^
0.90	2.74 × 10^15^	3.85 × 10^14^	2.25 × 10^16^	9.71 × 10^8^	2.37 × 10^9^	6.27 × 10^10^
0.95	2.30 × 10^15^	1.77 × 10^14^	2.29 × 10^16^	4.12 × 10^8^	1.18 × 10^9^	7.46 × 10^10^

**Table 7:** The activation energy, pre-exponential factor, and thermodynamic parameters obtained using the KAS method at a 10 °C/min heating rate for host and EPDM rubber loaded with 100 phr lead.

	EPDM 100 Lead at Rate 10 °C/min					EPDM 0 phr Lead at Rate 10 °C/min				
α	E	A	ΔH	ΔG	ΔS	E	A	ΔH	ΔG	ΔS
	(kJ/mol)	(sec^−1^)	(kJ/mol)	(kJ/mol)	(J/mol)	(kJ/mol)	(sec^−1^)	(kJ/mol)	(kJ/mol)	(J/mol)
0.05	206	1.47 × 10^12^	199.7	220.4	−27.6	162	3.51 × 10^9^	155.8	211.6	−77.5
0.1	217	1.01 × 10^13^	211.3	220.0	−11.6	161	3.13 × 10^9^	155.1	211.6	−78.6
0.15	221	2.01 × 10^13^	215.5	219.9	−5.9	161	3.20 × 10^9^	155.1	211.6	−78.5
0.2	224	2.84 × 10^13^	217.5	219.9	−3.1	160	2.60 × 10^9^	153.9	211.6	−80.4
0.25	225	3.59 × 10^13^	218.9	219.8	−1.2	159	2.10 × 10^9^	152.6	211.7	−82.2
0.3	225	3.49 × 10^13^	218.7	219.8	−1.5	156	1.33 × 10^9^	149.9	211.8	−86.0
0.35	225	3.33 × 10^13^	218.4	219.8	−1.9	155	1.01 × 10^9^	148.3	211.8	−88.4
0.4	224	3.12 × 10^13^	218.0	219.8	−2.5	154	9.41 × 10^8^	147.9	211.8	−89.0
0.45	224	2.82 × 10^13^	217.4	219.9	−3.3	153	7.72 × 10^8^	146.7	211.9	−90.7
0.5	223	2.72 × 10^13^	217.1	219.9	−3.7	153	7.60 × 10^8^	146.6	211.9	−90.8
0.55	224	3.23 × 10^13^	218.2	219.8	−2.2	153	7.62 × 10^8^	146.6	211.9	−90.8
0.6	223	2.43 × 10^13^	216.4	219.9	−4.6	153	7.15 × 10^8^	146.2	211.9	−91.4
0.65	224	2.80 × 10^13^	217.3	219.9	−3.4	153	7.20 × 10^8^	146.3	211.9	−91.3
0.7	223	2.61 × 10^13^	216.9	219.9	−4.0	152	6.85 × 10^8^	146.0	211.9	−91.8
0.75	222	2.28 × 10^13^	216.0	219.9	−5.2	153	7.50 × 10^8^	146.5	211.9	−91.0
0.8	223	2.68 × 10^13^	217.0	219.9	−3.8	152	7.00 × 10^8^	146.1	211.9	−91.6
0.85	223	2.45 × 10^13^	216.4	219.9	−4.6	152	6.77 × 10^8^	145.8	211.9	−91.9
0.9	222	2.26 × 10^13^	215.9	219.9	−5.3	152	6.94 × 10^8^	146.0	211.9	−91.8
0.95	223	2.73 × 10^13^	217.0	219.9	−3.8	153	7.22 × 10^8^	155.8	211.6	−77.5

**Table 8:** The activation energy, pre-exponential factor, and thermodynamic parameters obtained using the FWO method at a 10 °C/min heating rate for host and 100 phr loaded EPDM rubber.

FWO	EPDM 0 phr Lead at Rate 10 °C/min					EPDM 100 phr Lead at Rate 10 °C/min				
α	E	A	ΔH	ΔG	ΔS	E	A	ΔH	ΔG	ΔS
	(kj/mol)	(sec^−1^)	(kJ/mol)	(kJ/mol)	(J/mol)	(kj/mol)	(sec^−1^)	(kJ/mol)	(kJ/mol)	(j/mol)
0.05	210.0	3.0 × 10^12^	204.1	220.3	−21.5	164.5	5.74 × 10^9^	158.7	211.4	−73.5
0.1	223.3	2.7 × 10^13^	217.3	219.9	−3.4	166.0	7.48 × 10^9^	160.1	211.4	−71.4
0.15	228.3	6.2 × 10^13^	222.3	219.7	3.4	164.2	5.46 × 10^9^	158.2	211.5	−74.1
0.2	231.1	9.9 × 10^13^	225.1	219.7	7.2	163.6	4.92 × 10^9^	157.6	211.5	−75.0
0.25	231.9	1.1 × 10^14^	225.8	219.6	8.2	161.6	3.52 × 10^9^	155.6	211.6	−77.9
0.3	231.5	1.0 × 10^14^	225.4	219.6	7.7	160.1	2.71 × 10^9^	154.0	211.6	−80.1
0.35	231.6	1.1 × 10^14^	225.4	219.6	7.7	158.8	2.17 × 10^9^	152.7	211.7	−82.0
0.4	231.9	1.1 × 10^14^	225.7	219.6	8.1	157.9	1.83 × 10^9^	151.7	211.7	−83.5
0.45	231.5	1.0 × 10^14^	225.3	219.6	7.5	157.1	1.61 × 10^9^	151.0	211.7	−84.5
0.5	232.1	1.1 × 10^14^	225.9	219.6	8.3	157.0	1.57 × 10^9^	150.8	211.7	−84.8
0.55	231.7	1.1 × 10^14^	225.5	219.6	7.8	156.9	1.54 × 10^9^	150.7	211.7	−85.0
0.6	231.7	1.1 × 10^14^	225.4	219.6	7.7	156.1	1.34 × 10^9^	149.9	211.8	−86.1
0.65	231.0	9.6 × 10^13^	224.8	219.7	6.8	156.8	1.52 × 10^9^	150.6	211.7	−85.1
0.7	230.4	8.8 × 10^13^	224.2	219.7	6.0	156.0	1.33 × 10^9^	149.8	211.8	−86.3
0.75	230.6	9.1 × 10^13^	224.4	219.7	6.3	156.5	1.45 × 10^9^	150.3	211.7	−85.5
0.8	230.7	9.1 × 10^13^	224.4	219.7	6.3	156.6	1.46 × 10^9^	150.3	211.7	−85.5
0.85	230.5	8.8 × 10^13^	224.2	219.7	6.0	156.5	1.43 × 10^9^	150.2	211.7	−85.7
0.9	231.5	1.0 × 10^14^	225.2	219.6	7.4	157.2	1.64 × 10^9^	150.9	211.7	−84.6
0.95	231.6	1.1 × 10^14^	225.3	219.6	7.6	158.2	1.95 × 10^9^	151.9	211.7	−83.2

**Table 9:** The activation energy, pre-exponential factor, and thermodynamic parameters obtained using the Friedman method at a 10 °C/min heating rate for host and 100phr loaded EPDM rubber.

	EPDM 0 phr Lead at Rate 10 °C/min					EPDM 100 phr Lead at Rate 10 °C/min				
α	E	A	ΔH	ΔG	ΔS	E	A	ΔH	ΔG	ΔS
	(kJ/mol)	(sec^−1^)	(kJ/mol)	(kJ/mol)	(J/mol)	(kJ/mol)	(sec^−1^)	(kJ/mol)	(kJ/mol)	(J/mol)
0.05	245.5	1.0 × 10^15^	239.5	219.3	27.0	147.1	2.8 × 10^8^	141.3	212.1	−98.5
0.1	240.1	4.3 × 10^14^	234.1	219.4	19.5	150.0	4.7 × 10^8^	144.1	212.0	−94.5
0.15	237.8	2.9 × 10^14^	231.7	219.5	16.3	149.1	4.0 × 10^8^	143.1	212.0	−95.9
0.2	236.0	2.2 × 10^14^	229.9	219.5	13.8	145.6	2.2 × 10^8^	139.6	212.2	−101.1
0.25	231.4	1.0 × 10^14^	225.3	219.6	7.5	142.1	1.2 × 10^8^	136.0	212.3	−106.1
0.3	229.7	7.7 × 10^13^	223.6	219.7	5.1	139.3	7.2 × 10^7^	133.2	212.4	−110.3
0.35	226.9	4.9 × 10^13^	220.7	219.8	1.3	142.7	1.3 × 10^8^	136.6	212.3	−105.3
0.4	228.5	6.4 × 10^13^	222.4	219.7	3.5	146.6	2.6 × 10^8^	140.4	212.1	−99.8
0.45	227.4	5.3 × 10^13^	221.2	219.8	2.0	149.2	4.1 × 10^8^	143.1	212.0	−96.0
0.5	229.8	7.9 × 10^13^	223.6	219.7	5.2	149.9	4.5 × 10^8^	143.7	212.0	−95.1
0.55	228.0	5.9 × 10^13^	221.8	219.7	2.8	150.0	4.6 × 10^8^	143.8	212.0	−95.0
0.6	225.3	3.8 × 10^13^	219.1	219.8	−1.0	151.1	5.7 × 10^8^	144.9	212.0	−93.3
0.65	223.8	2.9 × 10^13^	217.5	219.9	−3.1	150.6	5.1 × 10^8^	144.3	212.0	−94.1
0.7	224.9	3.5 × 10^13^	218.6	219.8	−1.6	151.7	6.2 × 10^8^	145.4	211.9	−92.6
0.75	225.2	3.7 × 10^13^	219.0	219.8	−1.1	152.3	6.9 × 10^8^	146.0	211.9	−91.7
0.8	227.1	5.1 × 10^13^	220.9	219.8	1.5	152.3	6.9 × 10^8^	146.0	211.9	−91.7
0.85	226.4	4.5 × 10^13^	220.1	219.8	0.4	152.8	7.6 × 10^8^	146.5	211.9	−91.0
0.9	230.0	8.2 × 10^13^	223.7	219.7	5.4	155.6	1.2 × 10^9^	149.3	211.8	−86.9
0.95	254.2	4.3 × 10^15^	247.9	219.1	38.4	157.1	1.6 × 10^9^	150.7	211.7	−84.9

**Table 10:** Summary of the maximum, minimum, and average values of thermodynamic parameters (enthalpy change, Gibbs free energy change, and entropy change), activation energies (E), and pre-exponential factors (A).

		EPDM 0 phr Lead at Rate 10 °C/min					EPDM 100 phr Lead at Rate 10 °C/min				
KAS	Max	225.0	3.6 × 10^1^³	218.9	220.4	−1.2	162.0	3.5 × 10^9^	155.8	211.9	−77.5
	Min	206.0	1.5 × 10^12^	199.7	219.8	−27.6	152.0	6.8 × 10^8^	145.8	211.6	−91.9
	Average	222.2	2.6 × 10^1^³	216.0	219.9	−5.2	155.1	1.4 × 10^9^	149.3	211.8	−86.9
FWO	Max	232.1	1.1 × 10^14^	225.9	220.3	8.3	166.0	7.5 × 10^9^	160.1	211.8	−71.4
	Min	210.0	3.0 × 10^12^	204.1	219.6	−21.5	156.0	1.3 × 10^9^	149.8	211.4	−86.3
	Average	229.6	9.0 × 10^1^³	223.5	219.7	5.0	159.0	2.7 × 10^9^	152.9	211.6	−81.8
FM	Max	254.2	4.3 × 10^15^	247.9	219.9	38.4	157.1	1.6 × 10^9^	150.7	212.4	−84.9
	Min	223.8	2.9 × 10^1^³	217.5	219.1	−3.1	139.3	7.2 × 10^9^	133.2	211.7	−110.3
	Average	231.5	3.7 × 10^14^	225.3	219.7	7.5	149.2	5.2 × 10^9^	143.1	212.0	−96.0

## Data Availability

Detailed data supporting the findings of this study are included in the article, as the authors confirm. In any case, the original collected data are available on request from the corresponding author.
